# Comparison of the Effect of Scalp Block With Ropivacaine vs. Ropivacaine and Clonidine on Postoperative Pain in Patients Undergoing Craniotomy Surgery Under General Anesthesia

**DOI:** 10.7759/cureus.67342

**Published:** 2024-08-20

**Authors:** Aparna Bagle, Abhishek Raj, Ram Prakash B. U., Amala Kale

**Affiliations:** 1 Department of Anesthesiology, Dr. D. Y. Patil Medical College, Hospital and Research Centre, Dr. D. Y. Patil Vidyapeeth, Pune, IND; 2 Department of Community Medicine, Dr. D. Y. Patil Medical College, Hospital and Research Centre, Dr. D. Y. Patil Vidyapeeth, Pune, IND

**Keywords:** visual analog score, postoperative pain, ramsay sedation score, supratentorial craniotomy, scalp block, clonidine, ropivacaine

## Abstract

Introduction

Clonidine, an α2 agonist known for its hypotensive and analgesic effects, has proven beneficial in various routes of administration such as oral, intravenous, and local infiltration. Scalp blocks enhance hemodynamic stability during surgery and reduce intraoperative opioid requirements compared to controls in numerous studies. Additionally, they are effective in managing postoperative pain, resulting in reduced opioid consumption. Research has shown that clonidine can enhance and prolong the effects of intrathecal, epidural, and peripheral nerve blocks (e.g., brachial plexus, peribulbar). Here, we investigated the impact of adding clonidine at a dose of 1 μg/kg to scalp blocks performed with 0.5% ropivacaine for supra-tentorial craniotomy

Material and methods

This study was conducted on 60 patients under the American Society of Anesthesiologists (ASA) grade I and II who were scheduled for elective supratentorial craniotomy. Patients were divided into two equal groups of 30 and received a scalp block following general anesthesia. Patients in Group A (n=30) received a scalp block of 0.5% ropivacaine plus 1 ml of normal saline (total 21 cc). Patients in Group B (n=30) received a scalp block of 0.5% ropivacaine and clonidine (1 μg/kg) combined with 0.5 ml of normal saline (total 21 cc). Blood pressure, heart rate, peripheral oxygen saturation *(*SpO_2_), visual analog score, Ramsay sedation score, duration of analgesia, and analgesia requirement in the first 24 hours were recorded from baseline and postoperatively.

Results

The duration of first rescue analgesia for Group A was 4.30 ± 1.5 hours and that of Group B was 9.10 ± 1.4 hours. Duration of analgesia was significantly prolonged in patients receiving ropivacaine with clonidine for scalp nerve block. The amount of tramadol given in the first 24 hours in Group A, 62.50 ± 25.00 mg, was high compared to Group B, 57.14 ± 18.89 mg. The mean arterial blood pressure differed significantly in both groups at 30 minutes, 1 hour, 3 hours, and 12 hours after scalp block postoperatively and lower in Group B. Although changes in pulse rate, and SpO_2_ were not statistically significant in both groups, patients were hemodynamically stable and did not require any ionotropic support. Ramsay sedation score and visual analog score postoperatively were not significant. There were no significant adverse effects noted in any groups.

Conclusion

Our study concluded that administering clonidine at a dosage of 1 μg/kg, in combination with 0.5% ropivacaine for scalp nerve block procedures, significantly extends the duration of analgesia and enhances its quality, all while maintaining stable hemodynamic parameters.

## Introduction

The primary aim of anesthesia in neurosurgery is to prevent secondary brain injury that can occur due to factors like increased intracranial pressure, hypotension, or hypertension. Achieving excellent hemodynamic stability can be done through opioids, deeper anesthesia, or local anesthesia via infiltration or scalp nerve block (SNB). However, excessive use of opioids can lead to prolonged recovery and excessive blood pressure reduction when manipulation is minimal [1]. Combining general anesthesia with regional anesthesia has significantly improved intraoperative hemodynamics, surgical field, and postoperative pain management. Scalp blocks with local anesthetics (LAs) are being increasingly used alongside general anesthesia in neurosurgery to mitigate pin and incision responses and provide both intraoperative and postoperative analgesia. Numerous studies have shown that scalp blocks offer superior intraoperative hemodynamic stability and reduce intraoperative opioid needs compared to controls, with better postoperative pain management and reduced opioid consumption as well [2]. Clonidine, an α2 agonist, provides hypotensive and analgesic effects and is a versatile tool for anesthesiologists when administered orally, intravenously, or through infiltration. Studies have shown that clonidine enhances and prolongs the effects of intrathecal, epidural, and peripheral blocks like brachial plexus and peribulbar [3]. The reduction in the rate pressure product due to systemic absorption of clonidine can be advantageous in neuroanesthesia. Scalp blocks also offer preemptive analgesia, preventing physiological and neurological responses to noxious stimuli, thus reducing postoperative morbidity and mortality [3]. The advantages of preemptive analgesia include faster recovery, reduced endocrine stress response to surgery, decreased hyperglycemic response, improved respiratory function, early mobilization, and quicker discharge. Therefore, we designed a study to assess the effect of adding clonidine (1 μg/kg) to scalp blocks with 0.5% ropivacaine for supratentorial craniotomies under general anesthesia [4].

## Materials and methods

A prospective, single-blinded, randomized comparative study was conducted following approval from the Institutional Ethics Committee (IESC/411/2022) and registration with the Clinical Trials Registry-India (CTRI/2023/11/059907).

In a previous study by Wajekar, the time to first rescue analgesia was 408.17 ± 209.81 minutes in Group A and 887.97 ± 398.21 minutes in Group B. Considering this significant difference in analgesia duration, we used OpenEpi version 3 software (Open Source Epidemiologic Statistics for Public Health, www.OpenEpi.com) to calculate the sample size with a 95% confidence interval and 95% study power. The calculation suggested a total sample size of 24, with 12 participants in each group. However, to ensure better precision of the data, we decided to include a total sample size of 60 participants, with 30 in each group. Group A (n=30) received 0.5% ropivacaine plus 1 ml of normal saline (total 21 cc) in the scalp block, while Group B (n=30) received 0.5% ropivacaine and clonidine (1 μg/kg) plus 0.5 ml of normal saline (total 21 cc) in the scalp block [5].

A total of 60 patients with the American Society of Anesthesiologists (ASA) grade I and II, aged 18 to 65 years, Glasgow Coma Scale (GCS) 15/15, scheduled for supratentorial craniotomies, and surgery duration lasting less than 360 minutes were included in the study. Patients with uncontrolled hypertension or preoperative bradycardia, emergency cases, ischemic heart disease or cardiac arrhythmias, patients on alpha-blockers preoperatively, patients with raised intracranial tension (ICT), chronic alcoholism, and chronic drug abusers, severe pulmonary, hepatic or renal disease, history of craniotomy, drug allergy to LAs and/or clonidine, patient refused to participate in the study were excluded from the study.

Procedure and conduct of the study

Following a comprehensive pre-anesthetic examination that included a full history, clinical evaluation, and pertinent lab tests, the patients were chosen. Patients were randomly assigned to Group A and Group B using a computer-generated random number table and patients were kept nil by mouth six hours before the procedure. Anesthesia induction was carried out with midazolam 0.03 mg/kg, fentanyl 2 μg/kg, lignocaine 1 mg/kg, propofol 2 mg/kg, and vecuronium 0.1 and the patient was intubated with appropriate size endotracheal tube (ETT). Isoflurane 0.7 to 1.2 minimum alveolar concentration (MAC), a 50:50 combination of oxygen and nitrous oxide (N_2_O), and vecuronium top-up were used for maintenance. Immediately following intubation, a bilateral scalp block was administered using a 25-gauge needle to block the following nerves: the lesser and greater occipital on the line connecting the mastoid process and occipital protuberance; the zygomatic temporal nerve 1 cm from the outer canthus of the eye; the supraorbital and supra trochlear nerves near the supraorbital groove; and the auriculotemporal nerve near the tragus. The patient received a study medicine that had already been produced based on their research group assignment. At the end of surgery, all patients were extubated using extubation criteria.

Postoperatively, hemodynamics (pulse rate and mean arterial pressure), visual analog scale, and Ramsay sedation score were monitored every hour for the first four hours, then every four hours for the next 24 hours.

Statistical analysis

All the cases were completed in the stipulated time. Data was collected, compiled, and tabulated. The comparison of quantitative data was done by unpaired student's “t” test and was expressed as mean ± SD. Qualitative parameters were analyzed by Chi-square test.

The p value of <0.05 was considered significant.

## Results

The study involved 60 patients, classified as ASA grade I and II, all scheduled for elective supratentorial craniotomy. These patients were randomly divided into two groups: Group A, consisting of 30 patients who received 0.5% ropivacaine alone, and Group B, consisting of 30 patients who received 0.5% ropivacaine combined with clonidine. The average age of patients in Group A was 37.4 ± 10.0 years, while in Group B, it was 38.7 ± 11.3 years. Regarding gender distribution, Group A had 40% female (12 patients) and 60% male (18 patients), whereas Group B had 36.7% female (11 patients) and 63.3% male (19 patients). These demographic and clinical characteristics are shown in Table 1. 

**Table 1:** Demographic characteristics of the participants *Statistically significant if p<0.05 ASA: American Society of Anesthesiologists

		Group A	Group B	P value
Age (years)		37.4 ±10.0	38.7 ± 11.30	0.7077
Sex	Female	12	11	0.7722
	Male	18	19	
Weight (kg)		61.5 ± 5.9	63.8 ± 6.10	0.3932
ASA grade (I/II)		23/7	18/12	0.2428

The Ramsay sedation scores were comparable between Group A and Group B at baseline and various postoperative intervals up to 24 hours. Although Group A showed slightly higher baseline scores, the subsequent scores remained consistent for both groups. The p values above 0.05 indicate no clinically or statistically significant difference in sedation levels between the groups, as shown in Table 2.

**Table 2:** Ramsay sedation score in both groups *Statistically significant if p<0.05

Time	Group A	Group B	P value
Baseline Ramsay sedation score	1.93 ± 0.521	1.77 ± 0.504	0.213
Ramsay sedation score postoperatively			
4 hr	2.33 ± 0.479	2.27 ± 0.450	0.581
8 hr	2.33 ± 0.479	2.27 ± 0.450	0.581
12 hr	2.33 ± 0.479	2.27 ± 0.450	0.581
16 hr	2.33 ± 0.479	2.27 ± 0.450	0.581
20 hr	2.33 ± 0.479	2.27 ± 0.450	0.581
24 hr	2.33 ± 0.479	2.27 ± 0.450	0.581

Our study compared postoperative visual analog scale between Group A and Group B at various time intervals (4 hours, 8 hours, 12 hours, 16 hours, 20 hours, and 24 hours). No statistically significant differences were found between the groups as all p values were above 0.05, indicating similar pain levels postoperatively in both groups (Table 3).

**Table 3:** Visual analog scale in both groups *Statistically significant if p<0.05

Time	Group A	Group B	P value
Baseline visual analog scale	4.13 ± 2.080	3.90 ± 1.626	0.630
Visual analog scale postoperatively			
4 hr	2.23 ± 1.040	2.30 ± 1.149	0.815
8 hr	1.73 ± 0.785	1.90 ± 0.995	0.474
12 hr	1.33 ± 0.547	1.43 ± 0.679	0.532
16 hr	1.33 ± 0.547	1.40 ± 0.675	0.676
20 hr	1.30 ± 0.535	1.27 ± 0.521	0.808
24 hr	1.27 ± 0.450	1.20 ± 0.407	0.509

**Figure 1:**
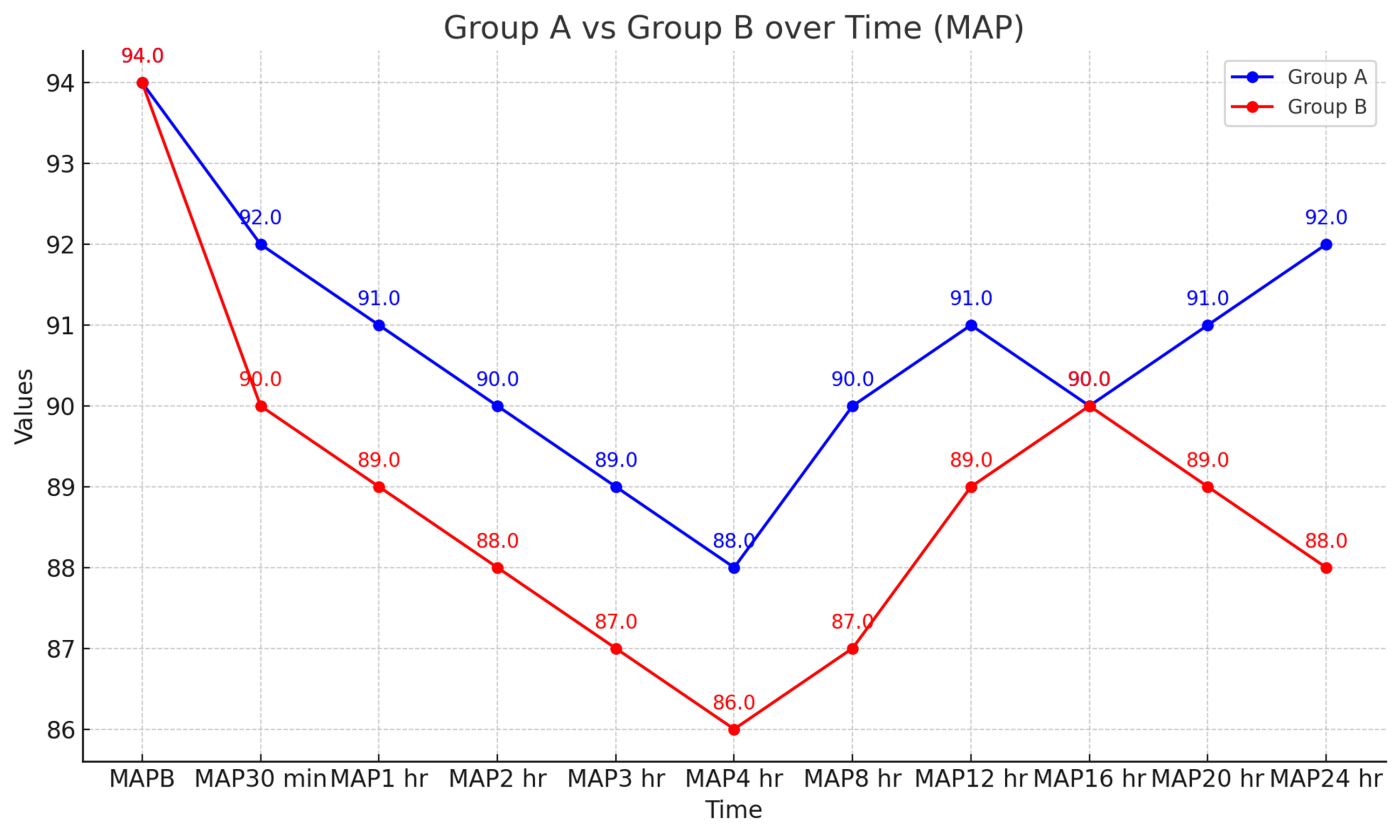
Comparison of mean arterial blood pressure (mmHg) MAPB: Mean arterial blood pressure at baseline; MAP: Mean arterial blood pressure

**Figure 2:**
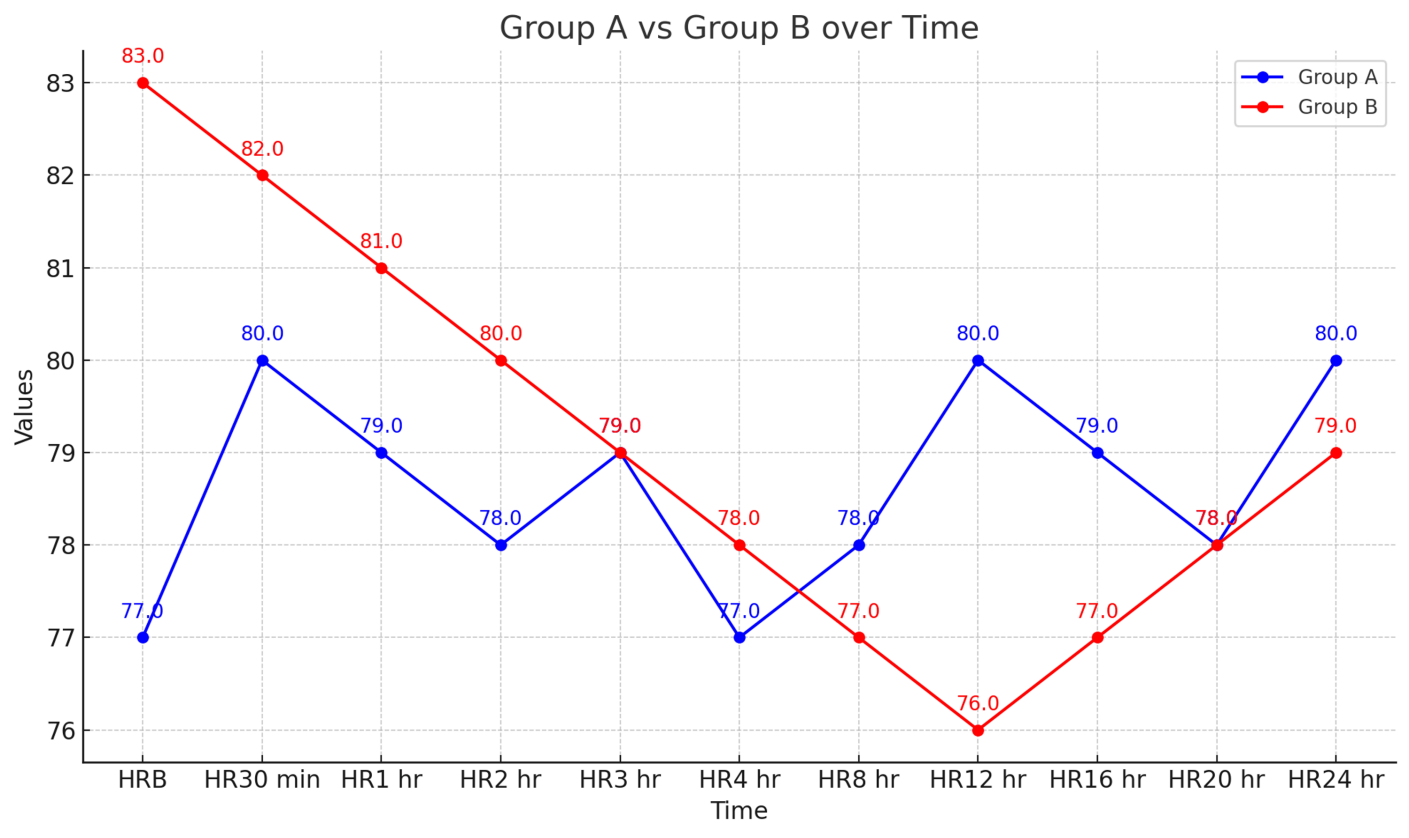
Comparison of pulse rates HR: Heart rate

Figure 1 and Figure 2 display hemodynamic parameters in both the study groups. Pulse rate was statistically significant at baseline, 4 hours, and 12 hours, but the difference was not clinically significant. Mean arterial blood pressure (MAP) changed significantly at 30 minutes, 1 hour, 3 hours, and 12 hours postoperatively, being lower in Group B, but these differences were not clinically significant. 

**Figure 3:**
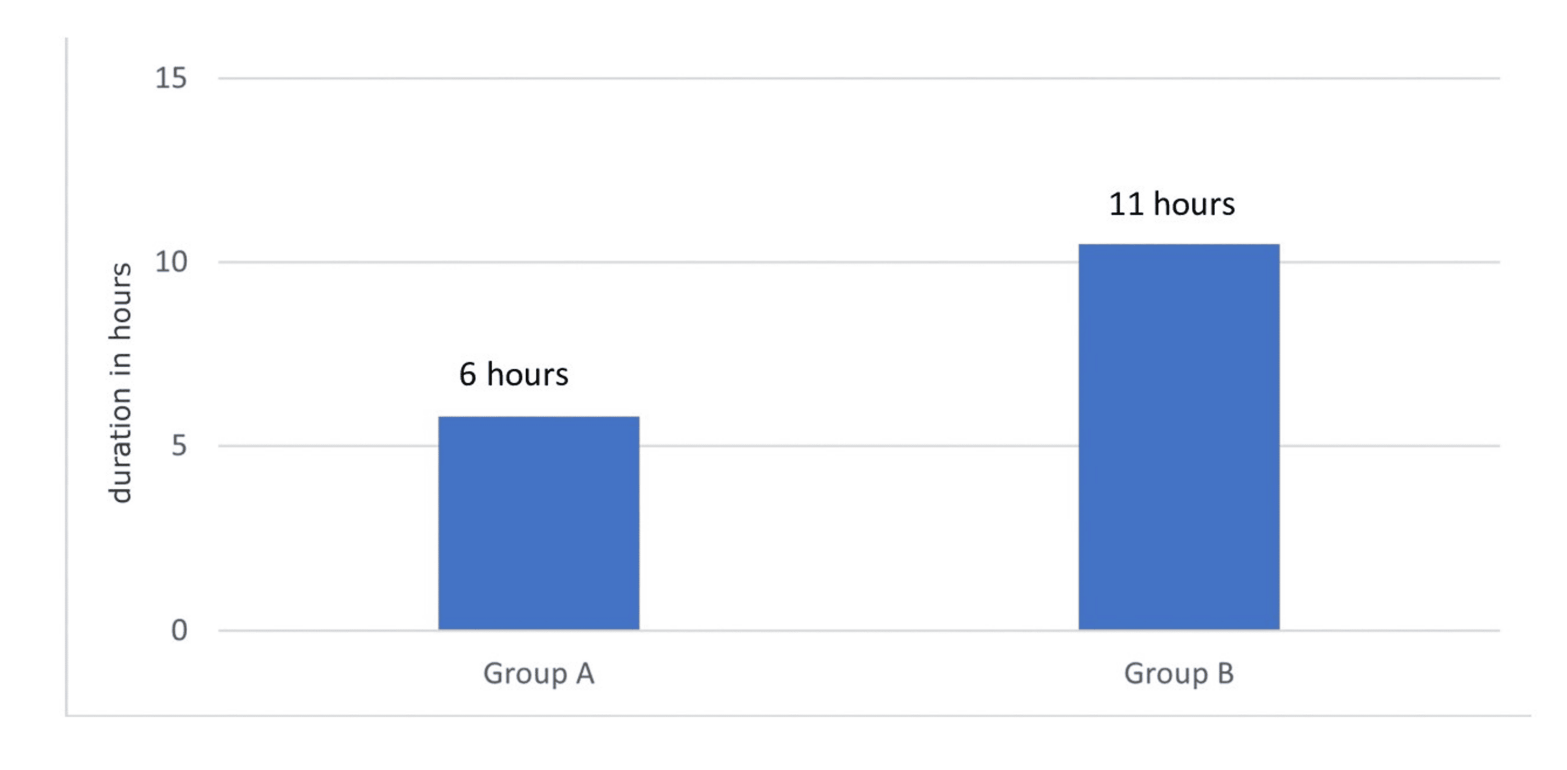
Mean duration of first rescue analgesia

When comparing the patients in Group A who were given just ropivacaine with those in Group B who were given ropivacaine together with clonidine, the mean length of the time to first rescue analgesia was considerably longer in Group B. The mean ± SD values of the initial rescue analgesia duration for each group, along with the corresponding p values, are displayed in Table 4 and Figure 3.

**Table 4:** Amount of tramadol given and time to first rescue analgesia in the first 24 hours in both groups *Statistically significant if p<0.05

Parameter	Group A	Group B	P value
Time to first rescue analgesia (hours)	4.3 ± 1.5	9.1 ± 1.4	<0.0001*
Amount of tramadol (mg) given in first 24 hours	62.50 ±25.00	57.14 ± 18.89	0.788*

The amount of tramadol (mg) given in the first 24 hours was higher in Group A (patients consuming only ropivacaine) compared to Group B (patients given ropivacaine with clonidine), though not significant statistically. The mean ± SD values of the amount of tramadol given in the first 24 hours in both groups are shown in Table 4, along with the respective p values.

## Discussion

Scalp block is a regional anesthetic technique that targets the sensory nerves of the scalp using long-acting LAs such as bupivacaine, ropivacaine, or levobupivacaine [6]. Ropivacaine, a long-acting amide LA developed as a pure enantiomer, works by reversibly inhibiting sodium ion influx in nerve fibers. It is less lipophilic than bupivacaine, leading to reduced motor blockade and a lower risk of cardiotoxicity and central nervous system toxicity, making it particularly suitable when a motor block is undesirable [7].

In our study, the demographic profile was similar across both groups. Group A, which received ropivacaine alone, had a time to first rescue analgesia of 4.30 ± 1.5 hours, while Group B, which received ropivacaine with clonidine, had a time of 9.10 ± 1.4 hours. The duration of analgesia was significantly longer in Group B, indicating that the addition of clonidine to ropivacaine prolongs analgesia. Furthermore, the total amount of tramadol administered in the first 24 hours was higher in Group A (62.50 ± 25.00 mg) compared to Group B (57.14 ± 18.89 mg). Mean arterial blood pressure measurements showed significant differences between the groups at 30 minutes, 1 hour, 3 hours, and 12 hours postoperatively, with lower readings in Group B. Although changes in heart rate and peripheral oxygen saturation (SpO_2_) were observed, these were not statistically significant, and patients in both groups remained hemodynamically stable without requiring ionotropic support. Postoperative Ramsay sedation scores and visual analog scores showed no significant differences between the groups, and no adverse effects were reported.

Nguyen et al. evaluated the efficacy of scalp block in reducing postoperative pain during brain surgery. In their study, 30 patients undergoing supratentorial craniotomy were randomly assigned to receive either 20 ml of 0.75% ropivacaine or 20 ml of 0.9% saline. The ropivacaine group experienced significantly lower average pain scores (2.0 ± 1.6) compared to the saline group (3.7 ± 2.4; P=0.036), although the total dose of codeine and the time to the first dose of codeine did not differ significantly between the groups [8].

Wajekar et al. investigated the effect of adding clonidine to 0.25% bupivacaine in scalp blocks for supratentorial craniotomies. Sixty patients were divided into two groups: Group A received 0.25% bupivacaine, while Group B received 0.25% bupivacaine with clonidine (2 μg/kg). Group B demonstrated significantly prolonged postoperative analgesia (887.97 ± 398.21 minutes) compared to Group A (408.17 ± 209.81 minutes; P<0.05) and better hemodynamic stability [5].

Yang et al. compared the effects of SNB and LA infiltration on inflammatory and hemodynamic responses and postoperative pain in craniotomy patients. Fifty-seven patients were divided into three groups: SNB with 15 ml of 0.75% ropivacaine, LA infiltration with 15 ml of 0.75% ropivacaine, and a control group receiving routine intravenous analgesia. The SNB group showed lower levels of interleukin 6 (IL-6) and C-reactive protein (CRP), higher levels of interleukin 10 (IL-10), and better pain control compared to the other groups [1].

Sahana et al. assessed the efficacy of adding dexmedetomidine to ropivacaine in scalp blocks for craniotomies. Sixty patients were divided into two groups: control (ropivacaine + saline) and experimental (ropivacaine + dexmedetomidine). Although both groups showed significant increases in heart rate and blood pressure post-skull pin insertion, dexmedetomidine did not provide significant additional benefits over ropivacaine alone [9].

Dash et al. examined the effects of clonidine on scalp block for supratentorial craniotomy. Sixty patients were divided into three groups: Group A (bupivacaine + saline + IV saline), Group B (bupivacaine + clonidine + IV saline), and Group C (bupivacaine + saline + IV clonidine). Clonidine in scalp blocks reduced propofol and fentanyl requirements and improved hemodynamic stability, providing prolonged postoperative analgesia [10].

The α2-adrenergic mechanism of analgesia has been studied for nearly a century, from the use of cocaine to the more recent use of dexmedetomidine and noradrenaline [11]. Clonidine targets α2 receptors in peripheral, spinal, and central nervous system regions, enhancing the effects of LAs in peripheral nerve blocks by acting on C and Aδ fibers. It increases transmembrane potassium conductance and reduces LA wash-out through vasoconstriction [12]. Since its topical introduction in 1984, clonidine has been recognized for its pharmacological effects beneficial to anesthesia, including sedation, hypnosis, analgesia, reduced opioid requirements, and attenuation of the sympathetic response to surgical stress [13]. Clonidine works by blocking adenyl-cyclase, decreasing intracellular cyclic adenosine monophosphate (cAMP), causing membrane hyperpolarization, and inhibiting voltage-dependent calcium channels. It has a higher affinity for α2 receptors compared to α1 receptors, making it a valuable adjunct in anesthetic practice [14,15].

## Conclusions

Our study concluded that clonidine, at a dosage of 1 µg/kg combined with 0.5% ropivacaine in SNB procedures, is an effective adjuvant. This combination significantly lengthens the duration of analgesia and enhances its quality without causing appreciable hemodynamic alterations. It is safe for patients undergoing supratentorial craniotomies and does not result in any adverse cardiovascular effects. Overall, the use of clonidine with ropivacaine provides an improved analgesic profile, making it a viable option for managing postoperative pain in these patients.

## References

[1] Yang X, Ma J, Li K (2019). A comparison of effects of scalp nerve block and local anesthetic infiltration on inflammatory response, hemodynamic response, and postoperative pain in patients undergoing craniotomy for cerebral aneurysms: a randomized controlled trial. BMC Anesthesiol.

[2] Maharani ND, Fuadi A, Halimi RA (2023). Comparison of the effect of scalp block analgesia bupivacaine 0.25% and clonidine 2 μg/kg with bupivacaine 0.25% and dexamethasone 8 mg on cortisol levels and Numeric Rating Scale in craniotomy tumour. Med J Malaysia.

[3] Sindou M, Leston J, Decullier E, Chapuis F (2007). Microvascular decompression for primary trigeminal neuralgia: long-term effectiveness and prognostic factors in a series of 362 consecutive patients with clear-cut neurovascular conflicts who underwent pure decompression. J Neurosurg.

[4] Panchal SM, Gosavi KS, Kudre KR (2023). Comparison of dexmedetomidine and clonidine as an adjuvant to bupivacaine in scalp block for supratentorial craniotomy surgery - a prospective, randomised Indian study. Int J Adv Res.

[5] Wajekar AS, Oak SP, Shetty AN, Jain RA (2016). A prospective, comparative, randomised, double blind study on the efficacy of addition of clonidine to 0.25% bupivacaine in scalp block for supratentorial craniotomies. Indian J Anaesth.

[6] Kuthiala G, Chaudhary G (2011). Ropivacaine: a review of its pharmacology and clinical use. Indian J Anaesth.

[7] Fernandes HS, Santos SA, Ashmawi HA (2018). Clonidine in anesthesiology: a brief review. Biomed J Sci Tech Res.

[8] Nguyen A, Girard F, Boudreault D (2001). Scalp nerve blocks decrease the severity of pain after craniotomy. Anesth Analg.

[9] Sahana BN, Radhapuram SD, Samantaray A, Hemanth N, Pasupuleti H, Mangu HR (2021). Comparison of effects of dexmedetomidine added to ropivacaine versus ropivacaine alone infiltration scalp block for attenuation of the haemodynamic response to skull pin placement in neurosurgical procedures: a double-blind, randomised clinical trial. Indian J Anaesth.

[10] Dash SK, Gosavi KS, Parikh HG, Kondwilkar B (2014). Effect of clonidine, by infiltration and by intravenous route, on scalp block for supratentorial craniotomy. South Afr J Anaesth Analg.

[11] Bijur PE, Silver W, Gallagher EJ (2001). Reliability of the visual analog scale for measurement of acute pain. Acad Emerg Med.

[12] Scott-Warren VL, Sebastian J (2016). Dexmedetomidine: its use in intensive care medicine and anaesthesia. BJA Educ.

[13] Vallapu S, Panda NB, Samagh N, Bharti N (2018). Efficacy of dexmedetomidine as an adjuvant to local anesthetic agent in scalp block and scalp infiltration to control postcraniotomy pain: a double-blind randomized trial. J Neurosci Rural Pract.

[14] Boussofara M, Mtaallah MH, Nefaa MN, Kaddour C (2004). Clonidine and anesthesia (Article in French). Tunis Med.

[15] Lee EK, Lee S, Kwon JH (2023). The efficacy of scalp nerve block in postoperative pain management after microvascular decompression: a randomized clinical trial. J Clin Med.

